# 25, 50 & 75 years ago

**DOI:** 10.1111/ans.70048

**Published:** 2025-02-22

**Authors:** Julian A. Smith

**Affiliations:** ^1^ Department of Surgery Monash University Melbourne Victoria Australia

## Twenty‐five years ago

**Collopy BT, Rodgers L, Woodruff P, Williams J. Early experience with clinical indicators in surgery. ANZ J. Surg. 2000;70:448–51**.

In 1997, a set of 53 clinical indicators developed by the Royal Australasian College of Surgeons (RACS) and the Australian Council on Healthcare Standards (ACHS) Care Evaluation Programme (CEP) was introduced into the ACHS Evaluation and Quality Improvement Programme (EQuIP). The clinical indicators covered 20 different conditions or procedures for eight speciality groups and were designed to act as flags to possible problems in surgical care. The development process took several years and included a literature review, field‐testing and revision of the indicators prior to approval by the College Council. In their first year, 155 healthcare organizations (HCO) addressed the indicators and this rose to 210 in 1998. Data were received from all states and both public and private facilities. The collected data for 1997 and 1998 for some of the indicators revealed rates, which were comparable with those reported in the international literature. For example, the rates of bile duct injury in laparoscopic cholecystectomy were 0.7% and 0.53%, respectively; the mortality rates for coronary artery graft surgery were 2.5% and 2.1%, respectively; the mortality rates after elective abdominal aortic aneurysm repair were 2.5% and 3.7%, respectively; and the post‐tonsillectomy reactionary haemorrhage rates were 0.9% and 1.3%, respectively. Results for some indicators differed appreciably from other reports, flagging the need for further investigation; for example, the negative histology rates for appendectomy in children were 18.6% and 21.2%, respectively, and the rates for completeness of excision of malignant skin tumours were 90.7% and 90%, respectively. The significance of these figures, however, depends upon validation of the data and their reliability and reproducibility. Because reliability can be finally determined only at the hospital level, they are of limited value for broader comparison. The process of review established for the indicator set has led to refinement of some indicators through improvement of definitions, and to a considerable reduction in the number of indicators to 29 (covering 18 procedures), for the second version of the indicators (which was introduced for use from January 1999). The clinical indicator programme, as it has with other disciplines, hopefully, will provide a stimulus to the modification and improvement of surgical practice. Clinician ownership should enhance the collection of reliable data and hence their usefulness.

**Foulds KA, Beasley SW, Moate K. Factors that influence length of stay after appendicectomy in children. ANZ J Surg 2000;70:43–6**.

The length of hospital stay following appendicectomy in children at Christchurch Hospital has decreased in recent years. The aim of the present study was to identify those factors that contributed to this change. A retrospective review of children admitted to Christchurch Hospital between 1994 and 1998 inclusive who underwent appendicectomy for suspected appendicitis was conducted. Data recorded included standard demographic information, symptom duration, operative details, analgesia, antibiotics, pathology, complications and postoperative length of stay (LOS). Postoperative LOS decreased significantly during the period reviewed across all degrees of appendiceal inflammation, from a mean of 70.5 to 50.1 h. The main determinant of postoperative hospital stay was the severity of the appendiceal inflammatory process.

Other factors that influenced LOS included surgical approach (open vs. laparoscopic), use of intra‐operative local anaesthesia, type and mode of postoperative analgesia, and age of the child. Longer duration of antibiotic use and symptom duration of greater than 24 h were associated with a longer LOS, primarily as a reflection of the severity of inflammation of the appendix. Factors that appeared to have little or no influence included gender and the experience of the surgeon. The severity of the inflammatory process appeared to be the main determinant of postoperative hospital LOS; advanced appendicitis with abscess formation or peritonitis was associated with the longest LOS, irrespective of the surgical approach, although the LOS after appendicectomy was reduced by a laparoscopic approach. Intraoperative local anaesthesia during open appendicectomy reduced hospital stay, probably because it reduced the need for postoperative narcotics. Early diagnosis (<24 h) was associated with a shorter postoperative LOS for acutely inflamed appendices.

## Fifty years ago

**Halliday P. The surgical management of subphrenic abscess: a historical study. ANZ J. Surg. 1975;45:235–44**.

This paper presents a sequential study of the development of the surgical management of subphrenic abscess from the earliest reports to the present day. Following the initial clinical recognition of subphrenic abscess of whatever aetiology, effective treatment depended on the patients' capacity to survive the initial peritonitis and to localize the infection as an abscess. During the years before the introduction of antibiotics, the classical clinical features of this condition were established and recorded in many series. Surgical techniques progressively evolved which permitted adequate drainage without undue dissemination of sepsis. In spite of improved diagnosis and improved surgical techniques, the average mortality rate after drainage remained unchanged except in the most expert hands. Following the introduction of antibiotics, hopes rose that mortality rates would fall significantly. Though the mortality of general peritonitis declined, clinical problems associated with subphrenic abscess increased greatly in complexity. The frequency of the more severe clinical manifestations lessened as a result of this therapy, so that, despite the introduction of special methods of investigation, a definitive diagnosis became not infrequently more difficult to make, in relation not only to the presence of an abscess, but also to its precise location. Extraserous drainage became widely accepted as the safest and most effective method of drainage prior to the introduction of antibiotics. As a result of the current increase in the difficulties of diagnosis, surgical opinion has moved to a position where all modes of exploration have advocates, with results that do not always justify the methods used. Extraserous drainage remains the safest method provided that the abscess can be located, and preliminary transpleural exploration appears for some abscesses to offer satisfactory solutions not only to problems of location, but also to problems of thoracic complications. The situation is capable of further improvement through recognition of the nature of the causal lesion. If this can be identified as a continuing source of infection, treatment of a related subphrenic abscess must include this factor as an urgent requirement.

**Wilson WF, Wilkie RC, Ewing MR. Pancreatic cyst complicated by major arterial erosion and gastrointestinal haemorrhage. ANZ J. Surg. 1975;45:85–90**.

**Fig. 1:**
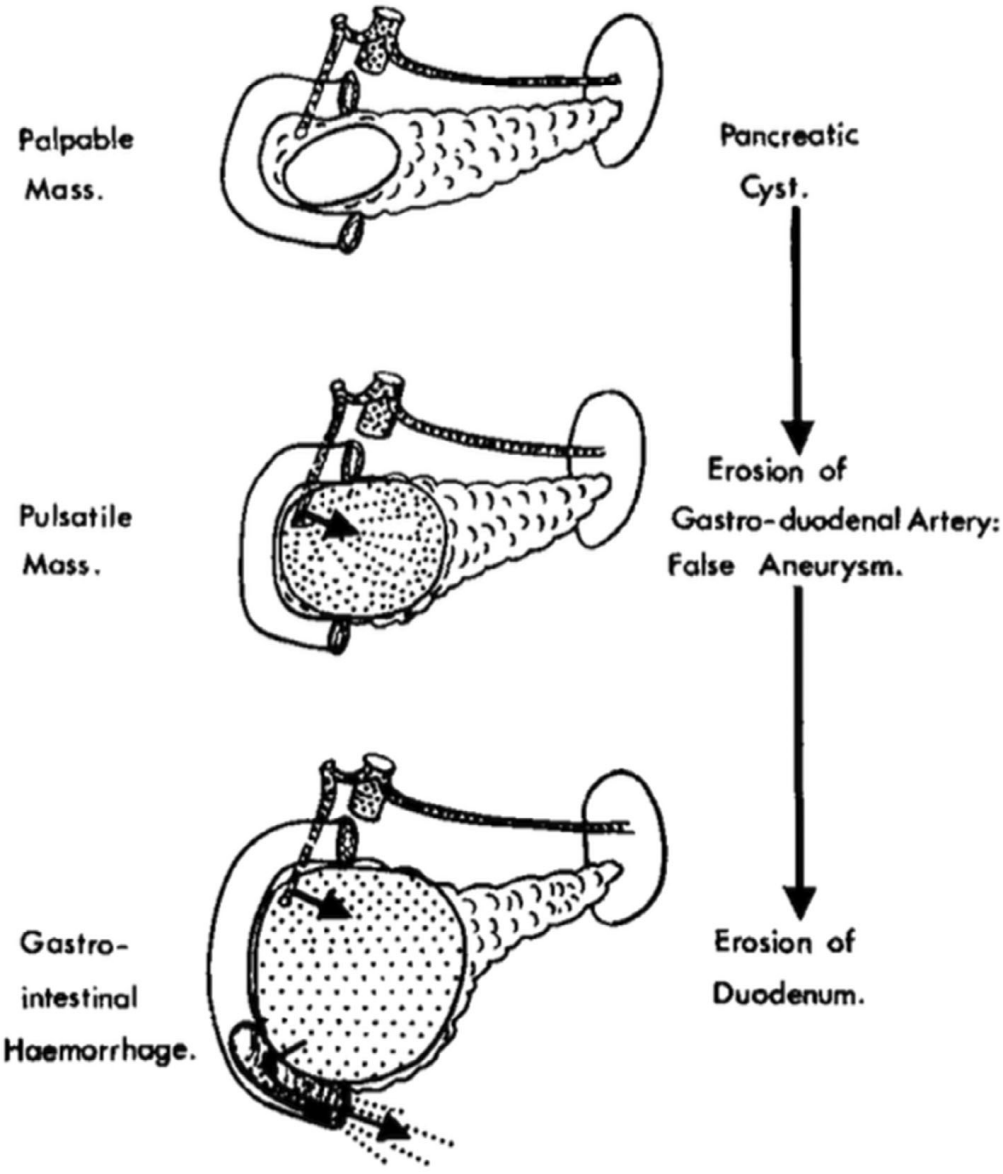
Diagrammatic representation of the stages in the development of the pathology.

Uncommonly, pancreatic cysts are complicated by the erosion of certain adjacent arteries and serious gastrointestinal haemorrhage (Fig. 1). This diagnosis should be entertained in any patient with chronic pancreatitis who presents with unexplained gastrointestinal blood loss, whether acute or chronic, a pulsatile mass in the epigastrium, and an associated bruit. Selective coeliac axis angiography may not only confirm the diagnosis but provide precise anatomical information as a guide to the surgeon in planning treatment. The surgical treatment of choice is transcystic ligation of the bleeding vessel, followed by internal cyst drainage.

## Seventy‐five years ago

**Sunderland S, Smith GK. The relative merits of various suture materials for the repair of severed nerves. ANZ J. Surg. 1950;20:85–113**.

In summary:

1. The results have been described as an experimental investigation undertaken to determine the relative merits of a number of different suture materials for the repair of severed nerves.
2. Fine plain catgut, white silk and human hair are the most suitable materials for repairing nerves. Of these three, silk has a slight advantage over plain catgut when technical factors as well as those associated with the reaction induced by the suture material are taken into consideration.
3. The outcome following nerve suture does not depend solely on the type of suture material employed but to a large degree on the manner in which it is used to unite the nerve ends. The principles which should govern the method of application of sutures in peripheral nerve surgery have been detailed.

